# The Structural Characteristics, Management, and Challenges of Backyard Poultry Farming in Residential Areas of Turkey

**DOI:** 10.3390/ani10122336

**Published:** 2020-12-09

**Authors:** Demir Özdemir

**Affiliations:** Department of Agricultural Biotechnology, Faculty of Agriculture, Akdeniz University, 07058 Antalya, Turkey; dozdemir@akdeniz.edu.tr; Tel.: +90-505-818-1279

**Keywords:** backyard poultry, flock management, biosecurity measures, challenges, survey

## Abstract

**Simple Summary:**

Relatively little is known about backyard poultry flocks in urban areas in Turkey, their size and how they are managed. To address this knowledge gap, a semi-structured survey was conducted of backyard poultry owners in Turkey with regard to flock characteristics, management, biosecurity practices and the challenges of backyard poultry flocks. Data derived from 1094 respondents across Turkey showed that the majority of respondents owned small flocks with fewer than 50 birds. Most participants reported keeping poultry as food for family use and free-range coops were the most preferred type of housing. Keeping different poultry species together, which poses a significant biosecurity risk, was reported by 64% of owners. Internal-external parasites, *Escherichia coli* infections and chronic respiratory diseases were the most common health problems in backyard flocks. Although there is a significant amount of backyard poultry farming activity in residential districts of Turkey, lack of regional regulations and animal theft were the most cited challenges. The results highlighted the need for biosecurity measures and for a regulatory framework that takes into account the risks to commercial poultry flocks and public health. A thorough understanding of the complexities of backyard poultry practices and the needs of backyard breeders will help authorities to design effective policies for the backyard poultry sector in Turkey.

**Abstract:**

The aim of this study was to collect, for the first time, comprehensive information about the backyard poultry sector in Turkey. This included a profile of the poultry owners, flock characteristics, husbandry, housing conditions, the owners’ biosecurity measures and the challenges of backyard poultry farming in residential areas of Turkey. An online semi-structured survey was fully completed by 1094 respondents. The majority of respondents (91%) owned fewer than 50 birds and reported raising other poultry species besides chickens (64%). Most of the participants indicated that they kept poultry as food for family use (83%) and had been involved in chicken-raising activities for less than 10 years (86%). Free-range coops were the type of housing most preferred by the respondents (86%). However, there was a lack of awareness about poultry diseases and poultry health care conditions. Respondents that confirmed wild bird and rodent access to their feeders and drinkers reported high rates of internal-external parasites, *Escherichia coli* infections and chronic respiratory diseases (*p* < 0.001). Lack of regional regulations (84%), animal theft (80%), lack of information on poultry management (79%), minimizing predation (75%), and the need for vaccination and veterinary services (73%) were the most cited challenges. The results highlighted the need for improved biosecurity measures and for a regulatory framework that takes into account the risks to commercial poultry flocks and public health.

## 1. Introduction

Backyard poultry farming is regarded as a valuable tool for enhancing the socio-economic and nutritional status of people in rural areas by providing cheap sources of protein and additional employment [1]. In addition to its contribution to the countryside, raising poultry in backyards has recently become very popular in urban areas [2,3,4]. The key reasons for keeping poultry in residential backyards are consumer demand for nutritious and fresh food [5,6,7], it provides extra revenue [2], concerns about animal health and welfare, and chickens are raised as pets or a hobby [2,6,8]. Despite its positive aspects, backyard poultry farming activities in urban areas are seen as a risk factor in terms of public health and for commercial poultry flocks because of their interactions with wildlife and their epidemic potential. This is why the activities of backyard poultry have been studied in a multi-dimensional way, such as in relation to husbandry methods, poultry health and biosecurity at the international level [2,4,6,7,9,10].

The poultry industry has developed steadily in Turkey, and until recently, the country’s egg and white meat needs has been met by large-scale professionally-controlled intensive-processing plants that operate under strict biosecurity criteria. All phases of these industrial operations are registered by the government including where they are located, the number and capacity of the plants, and their flock management practices. However, it is clear that the same surveillance programs do not exist for backyard poultry flocks. Currently, the registration of backyard poultry flocks in Turkey is carried out voluntarily by a number of hobby and ornamental poultry associations; therefore, there is no accurate information about the number of owners, flock size, flock location or how they are managed. Furthermore, with the increasing prevalence of backyard poultry farming in urban settlements, regional authorities are often put in a challenging position with regard to the legal framework. Municipalities across the country are facing difficulties in controlling backyard poultry farming in residential areas since there is no enforceable law in Turkey that specifies the rules for raising backyard poultry.

In some countries, backyard poultry farming has been found to be associated with outbreaks of Newcastle disease, salmonella infections and avian influenza [11,12,13,14]. Similarly, Turkey experienced outbreaks of highly pathogenic avian influenza (H5N1) in 2006, and it was reported that the transmission of the H5N1 virus from wild animals to domestic animals in Turkey was closely connected to backyard poultry farming [15]. Therefore, there is a need to investigate the increasing practice of backyard poultry farming in urban settlements in Turkey because of its benefits as well as its risks to public health. The aim of this study was to collect, for the first time, detailed information about the poultry owners, flock characteristics, poultry-keeping practices, their housing conditions and owners’ knowledge of common poultry diseases and biosecurity practices in backyard poultry flocks in residential areas of Turkey.

## 2. Materials and Methods

### 2.1. Sample Frame

Preliminary details for the sample frame were gathered from the Turkish Ornamental Poultry and Garden Animals Federation (TSHF, Istanbul, Turkey), which is the only legal poultry breeders’ association in Turkey. According to interviews with TSHF officials, there are probably more than 10,000 backyard poultry breeders in Turkey, although they reported that they have more than 3000 registered members who keep poultry in their backyards. The scope of the study included TSHF member breeders and also focused on breeders across Turkey involved in backyard poultry farming. With this in mind, the survey was announced to backyard poultry breeders in Turkey through a television program, training seminars given by the TSHF, Facebook, Twitter and Google Groups. Following the announcement of the survey, a total of 3201 owners eventually agreed to participate in the survey. The survey results showed that 63% of respondents were not registered with any breeders’ association, and also that the survey reached 63% unregistered breeders who were not members of the TSHF.

### 2.2. Data Collection

A semi-structured questionnaire was developed by the author based on interviews with breeders, field observations and the relevant literature. The survey was tested in a pilot study with 49 breeders and it was revised according to the feedback obtained from these breeders. The revised version of the questionnaire comprised a total of 41 questions divided into five parts. The first part pertained to information about flock inventory (number of species, breeds); the second part pertained to the physical conditions of coops and husbandry practices; the third pertained to bird health, disease knowledge and biosecurity measures, feeding practices and welfare; the fourth part pertained to experience and motivation for keeping poultry, challenges, membership of any breeders’ association; and the fifth part related to participants’ demographics and expectations of authorities. Backyard poultry keepers are not registered in Turkey, so it was not possible to do a postal survey. Turkey has a very good internet infrastructure at the regional level, so the questionnaire was applied using SmartSurvey, an online survey software (https://www.smartsurvey.co.uk/). The survey was open from April 2019 to August 2019 and it was announced to potential participants through various communication channels. No personal identifiers such as names, addresses, race or ethnicity were collected as part of the demographic information.

### 2.3. Statistical Analysis

A total of 3201 breeders participated in the online survey, 2107 participants partially completed the survey and 1094 questionnaires were fully completed. Multiple entries from the same IP address were evaluated as a single participant and only fully completed surveys (1094) were analyzed. Hence, a total of 1094 questionnaires were included in the analysis. Descriptive statistics were generated for all the variables in the dataset. The Pearson chi-square or the likelihood ratio test was used to determine if there was any statistically significant difference between variables and for all tests the significance was defined as *p* < 0.05. Because not all of the questions were answered by each respondent, the overall number of answers obtained for each question was the denominator used in the calculations. Most questions in the survey were categorical and analyzed using frequency and multinomial logistic regression. For the multinomial logistic regression, five outcome variables were considered (reasons for keeping poultry, biosecurity awareness, source of information about poultry healthcare, use of veterinary services and challenges). Given that most of these variables were not binary, they were modified for use by combining categories into two per variable (yes/no). The living environment of the respondents was considered a dependent variable and included urban, sub-urban and rural, where urban was selected as reference and significance was defined as *p* < 0.05. The data were analyzed using SPSS (IBM Corp. SPSS Statistics Base v23. 2017, Armonk, NY, USA).

## 3. Results and Discussion

### 3.1. Respondents’ Demographics

**Figure 1:**
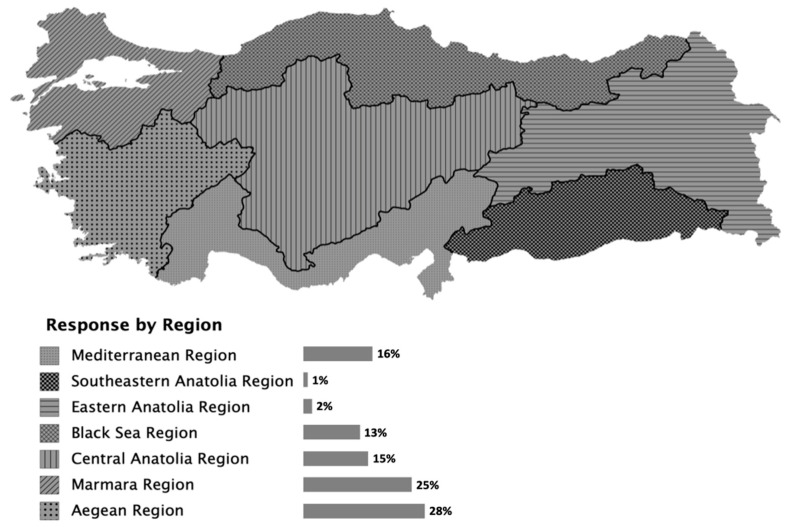
Distribution of survey participants by region.

From the city codes collected as a part of the demographic information, it was concluded that participants in the survey represented all of the regions in Turkey (Figure 1). As the type of area might differ within the same city code, respondents were asked to determine if the region they lived in was urban, sub-urban or rural. The results showed that 52.2% of the respondents described their living areas as urban, 24.2% as sub-urban, and 23.6% as rural. As in this research, backyard poultry raising has been found to be increasing steadily in urban areas in other countries [2,16,17,18].

**Figure 2:**
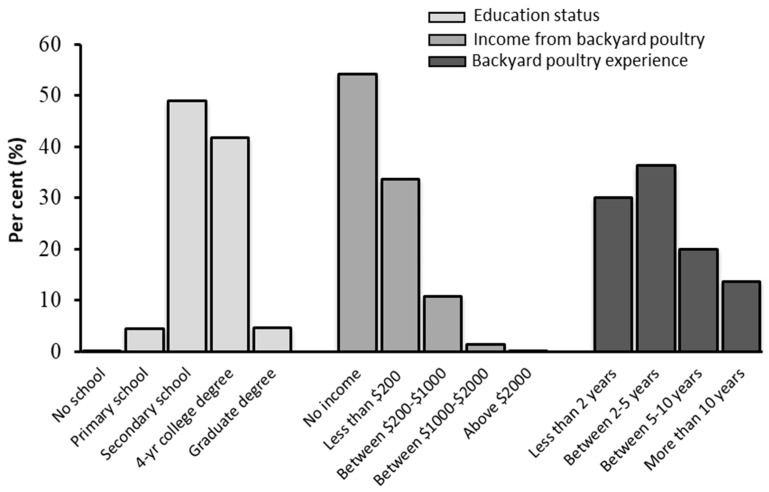
Demographic information for the survey participants.

Most of the respondents were younger than 50 years old (90%) and had been educated beyond secondary school level (95.4%). When participants were asked how much income they earned from backyard poultry farming, 54% stated that they did not raise poultry for the purpose of generating income, and the rest of the participants stated that they earned 200 USD/month and more from backyard poultry farming (Figure 2). This indicated that more than half of the participants were interested in backyard poultry for their own needs (poultry meat and eggs) and hobby purposes.

**Figure 3:**
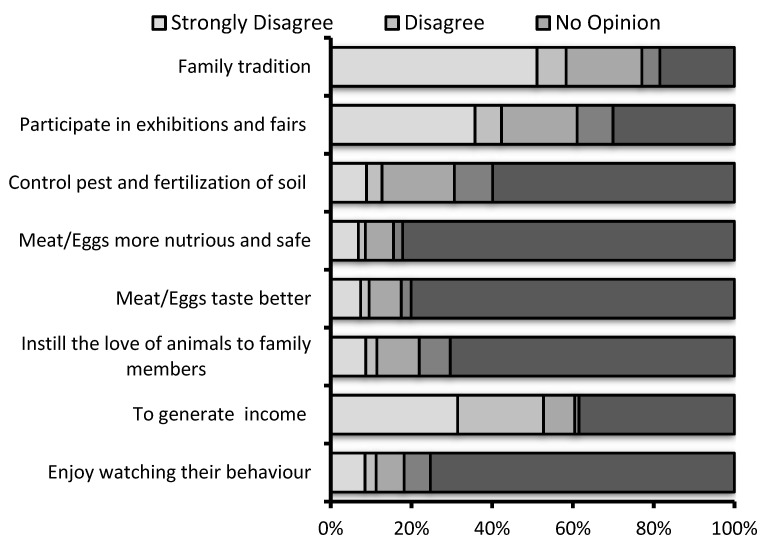
Backyard poultry owners’ reasons for keeping poultry.

The majority of the respondents (66%) indicated that they had kept poultry for less than 5 years (Figure 2), which confirms the increased interest in backyard poultry in recent years. According to the chi-square analysis, the relationship between the backyard poultry keeping experience and income per month from backyard poultry was significant (χ^2^ = 45.249; *p* < 0.001) and the results showed that respondents who had less than 5 years of experience in backyard poultry did not keep poultry for income purposes. The participants were requested to describe their reasons for raising poultry by rating how much they agreed with various statements on a Likert (1: strongly disagree and 5: strongly agree). The majority of the participants agreed that the meat and eggs obtained from backyard poultry was tastier (82.5%), healthier and more nutritious (84.5%) than commercial poultry products (Figure 3). Moreover, most of the respondents stated that they enjoyed watching the behavior of the poultry (81.8%), keeping poultry instilled a love of animals in family members (78.1%), and they helped to control pests and fertilized the soil in the garden (69.4%). Generating income from backyard poultry, participating in exhibitions and fairs, and family tradition was the less important to the participants. In summary, these findings confirmed that the participants perceived their poultry as pets and/or thought that the meat and eggs they obtained from their animals were healthier and better quality than commercial ones. Also, when the relationship between the education status of participants and the reasons for raising poultry in backyards was examined according to the chi-square analysis, it was concluded that respondents with higher education tended to raise poultry to obtain healthier, tastier and nutritious meat and eggs for their families (χ^2^ = 85.927; *p* < 0.0001).

The reason for keeping poultry in the backyard is associated with the living environment of respondents. Respondents living in sub-urban areas have 1.3 times (1/0.78) lower odds (*p* < 0.05) of keeping poultry to generate income compared to respondents living in urban areas, while rural respondents were 1.23 times more likely to keep poultry to generate income compared to respondents living in urban areas (Table 1). Backyard poultry owners’ top ranked reasons for keeping poultry in Turkey also scored highly in other studies in the USA [2,19], the UK [3,17], Australia [20] and Chile [9].

### 3.2. Flock Structure and Husbandry

Backyard poultry farming is regarded as an activity that contributes to poultry biodiversity due to its genetic richness [21], therefore, breeders were asked about their breed preference. More than half of the respondents (59%) indicated that they were keeping native chicken breeds while the others kept 48 chicken breeds of foreign-origin, with the most popular being the Brahma (24%) and Sussex varieties (18%). Furthermore, 64% of respondents stated that they also raised other poultry species such as quail (24%), geese (16%), duck (14%), and turkey (11%) besides chickens in their coops. It is widely accepted that raising different kinds of poultry species together increases the risk of cross-species transmission. Other studies have also reported that it is common for backyard poultry owners to keep other species of birds besides chickens [9,22,23,24].

Regarding the characterization of backyard flocks, the vast majority of the respondents (91%) indicated their flock size was less than 50 birds. Respondents who live in rural and sub-urban areas reported having larger flocks and described their poultry houses as sheds or coops with area varying from less than 10 m^2^ (43.8%), 10 to 50 m^2^ (33.2%) and over 50 m^2^ (23%). The majority of participants (85.9%) had free-range coops in which the chickens could behave naturally during the day. Free-range coops keep animal welfare at the forefront, and are also widely preferred by backyard poultry breeders in other countries [2,17]. Although free-range coops provide the chickens with freedom of movement, they require a range of measures to protect the chickens from predators. Therefore, respondents were asked about the precautions taken to prevent predation. Fencing around the poultry area was the most preferred (60.2%) method while others preferred closing the birds inside a shed to protect them against predators. With regard to feeding practices, the survey showed that breeders feed their poultry flocks with a variety of food. About 34.2% of owners purchased commercial feed, while others fed their flocks with kitchen scraps (23.1%), grain-based feed (22.1%) and self-formulated rations (16.4%). Moreover, 4.2% of respondents stated that they did not feed their poultry flocks and the birds eat whatever they find in the environment. Except for daily rations, 83.5% of breeders reported that they supplemented their chickens’ diet with additional synthetic vitamins and minerals. Although various feed sources are used in the diets of backyard chickens, kitchen scraps tend to be a preferred source of feed because they are easily accessible and cheap. However, feeding farm animals with kitchen scraps is thought to pose a risk of spreading disease. In Turkey, there is no legislation stating the rules on backyard poultry farming, so there is no restriction on feeding animals with kitchen scraps. However, it has been reported that in the UK, a range of bans on feeding chickens with kitchen scraps are enforced by the relevant legislation [3,17].

### 3.3. Biosecurity and Bird Health

**Figure 4:**
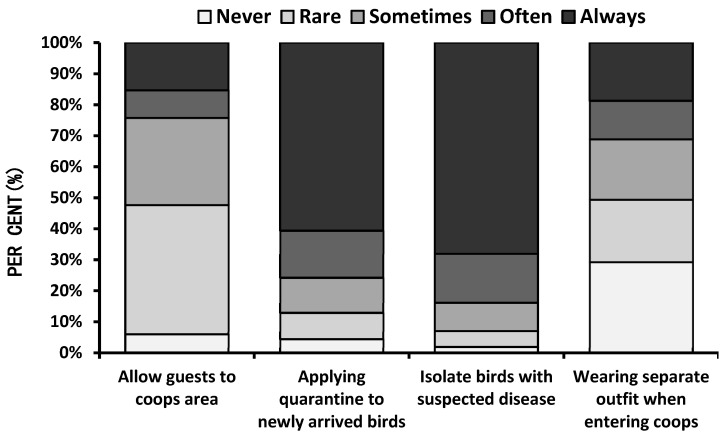
Biosecurity awareness of the survey respondents.

Regarding biosecurity measures for backyard poultry, respondents were asked how often they implemented any biosecurity practices, which were evaluated on a Likert preference scale (Figure 4). The majority of respondents always quarantined newly arrived chickens as well as birds with suspected diseases. However, most of the breeders did not wear protective clothing when entering coops. Nearly half of the respondents (47.7%) were sensitive about allowing visitors into the coop area while the rest were likely to allow guests. With respect to biosecurity measures, when asked about how often they clean their coop area, the most common replies were that they cleaned the coops weekly (54.4%), monthly (22.8%) and when needed (16.7%). However, 0.2% of respondents reported that they never cleaned their coops, while 4.9% and 1% of reported cleaning their poultry areas daily and once a year, respectively. To understand the risks of disease contamination, respondents were asked if wild birds and/or rodents have access to their chickens’ drinkers and feeders. While more than half (57.1%) of respondents denied that wild birds or rodents had access to drinker and feeder areas, 34.6% confirmed that wild birds/rodents had access, and 8.3% of the respondents had no information about access. Furthermore, when asked about the disposal of dead animals in terms of the potential to spread an infectious disease, respondents reported that they disposed of dead animals by burying (46.6%), trashing (46.2%) or burning (7.2%) them. Logistic regression results showed that biosecurity awareness was significantly associated with the respondents’ living environment. Respondents living in sub-urban areas were more likely to quarantine new animals compared to respondents living in urban areas (Odds ratio (OR) = 1.36, *p* < 0.01), while respondents in rural areas compared to respondents living in urban areas were less likely to quarantine new animals (OR = 0.70, *p* < 0.01) (Table 1). Although the findings demonstrated some degree of understanding of the need for biosafety practices, participants were unaware of the relationship between wild bird/rodent access to their coops or animal carcass disposal practices and the risks of transmission of diseases. Similar knowledge gaps in biosecurity practices in backyard poultry owners have been reported in other studies [2,3,9,23,25].

Participants were asked where they received support for their poultry health care. The results were quite varied with 50.9% responding that they got support from the internet, 29.9% from veterinarians, 7.6% from books and magazines, 3.3% from academic sources, 3.1% from agricultural agencies, 3.1% from feed dealers and 2.1% from friends. The living environment of respondents affected their choice of information source. Respondents from sub-urban areas were more likely to use the internet (OR = 4.79, *p* < 0.01) as a source of information about poultry health and were less likely to consult veterinary services (OR = 0.76, *p* < 0.001) (Table 1). Respondents were also asked when they used veterinary services; 40% of respondents stated that they had used veterinary services when they encountered disease in their animals, while 25.8% stated that they had never received veterinary support for their animals. Besides, other respondents stated that they got veterinary support periodically every six months (14.1%), monthly (13.7%) and once a year (5.5%). Respondents living in sub-urban areas were more likely to contact veterinary services when they encountered a disease compared to respondents living in urban areas (OR = 1.57, *p* < 0.01) (Table 1).

**Figure 5:**
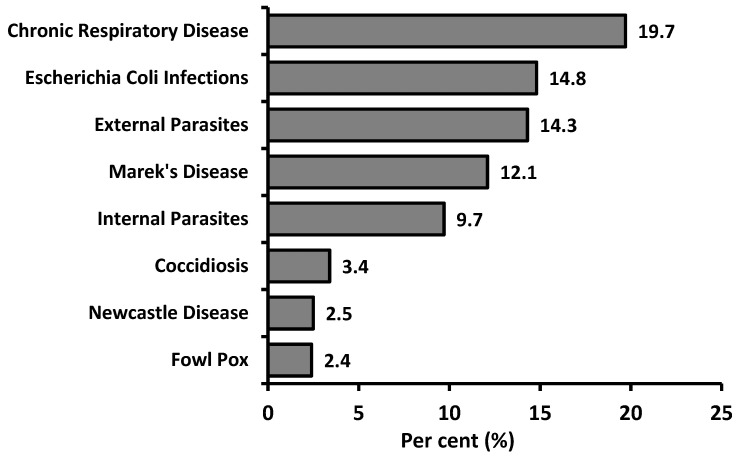
The most common poultry diseases reported by the respondents.

Backyard poultry farming is considered as a risk for epidemic diseases because of the low level of biosecurity measures. In this context, the most common diseases encountered by breeders in backyard poultry keeping were reported by the respondents and are shown in Figure 5. Although the results were variable, chronic respiratory disease (CRD) (19.7%) was reported as the most common disease. In addition, *Escherichia coli* infections (14.8%), external parasites (14.3%), Marek’s disease (12.1%) and internal parasites (9.7%) were reported by the participants as second-level diseases. Coccidiosis, Newcastle disease and fowl pox were the other diseases identified by the respondents. Similar to the findings of Crespo and Senties-Cue [26], highly transmissible poultry diseases such as avian influenza were not frequently reported in backyard poultry flocks; however, this result may also be related to participants’ level of knowledge and ability to recognize chicken diseases and symptoms. However, a significant association was found between the access of wild birds/rodents to the coop pen, and encountering disease (χ^2^ = 79.968; *p* < 0.001). The respondents that confirmed wild bird/rodent access to their feeders and drinkers reported higher rates of internal-external parasites, *Escherichia coli* infections and CRD.

### 3.4. Challenges

**Figure 6:**
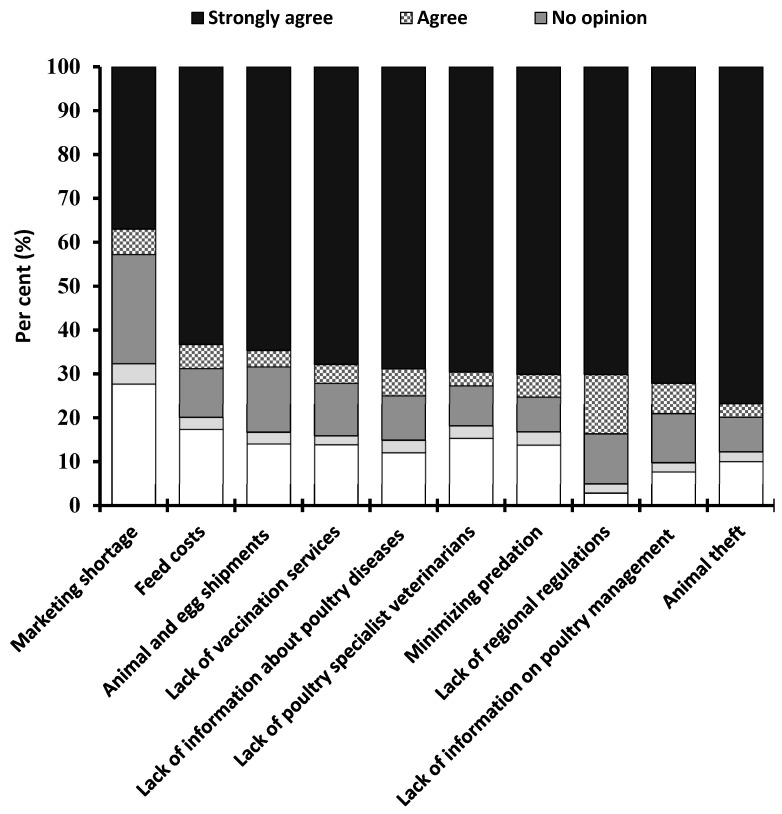
The major challenges reported by the respondents.

Finally, the participants were asked about the major challenges they faced in regard to backyard poultry keeping practices. Ten major challenges were identified and evaluated with a Likert scale (Figure 6). The vast majority of the respondents agreed with the following challenges: lack of regional regulations for backyard poultry (83.68%), animal theft incidents (79.89%), lack of information on poultry management (79.07%), minimizing predation (75.23%), lack of information about poultry diseases (74.95%) and lack of poultry specialist veterinary services (72.67%). The other important challenges were reported as the lack of vaccination services for small flocks (72.12%), feed costs (68.74%), animal and egg shipments (68.37%) and a shortage of marketing (62.98%). Moreover, the respondents’ living environment had a significant impact on the challenges they were facing (Table 1). Animal theft and the lack of backyard poultry farming legislation were of great concern to respondents from both sub-urban and rural areas. Individuals living in rural areas found preventing predation more challenging than respondents living in sub-urban areas (OR = 1.39, *p* < 0.001) (Table 1).

The need for legislation that is specific to backyard poultry was strongly supported by 83.68% of the survey participants. Most of these respondents lived in urban and sub-urban areas. The majority of people engaged in poultry activities in these regions had received complaints from their neighbors about the smell of manure, the noise of their chickens or concerns about zoonotic diseases. In case of such a complaint, due to lack of legislation regarding poultry keeping in residential areas, breeders may be fined by the municipal police and penalties such as confiscation of animals may be imposed. The regulation of poultry rearing in residential districts by legislation has still not been enacted in Turkey and people who want to raise chickens in urban residential areas are subject to the same sanctions adopted for commercial chicken flocks. Similarly, the lack of legislation was also reported to be highly challenging for backyard poultry owners in the United States [2,27] and the UK [17,28].

Although respondents reported keeping their poultry in a closed and sheltered area at night, they reported predation (75.23%) and animal theft (79.89%) as major problems. Notably, animal theft was more frequent in hobby and ornamental poultry coops, which consist of expensive and exotic birds. Predation and animal theft have also been reported as a major problem for backyard poultry owners in other studies [29,30]. Respondents also complained about poultry health and biosecurity issues, which were reflected in the ratings for the lack of trained veterinarians for treating chickens, the lack of information about poultry diseases and the lack of a vaccination schedule for small backyard flocks in Turkey. At the end of the survey, breeders were asked an open-ended question about their expectations of the authorities, and 78% of those who answered this question reported that they expected government support on poultry health, veterinary services and vaccination. As in many countries, backyard poultry farming in Turkey has a scattered structure with small-scale poultry flocks. Backyard flocks are not uniform, and therefore, it is not possible to actualize all-in-all-out systems in backyard coops as in commercial poultry flocks, which makes it difficult to synchronize veterinary services or a vaccination schedule for small-scale backyard poultry flocks in Turkey. In this context, ensuring the regional enrolment and organization of breeders would enable the synchronization of veterinary services such as vaccination.

## 4. Conclusions

While backyard poultry keeping has increased in popularity in Turkey’s residential areas over the past few years, there is not much information available on these activities at present. The current research is the first comprehensive attempt to identify the structural characteristics, management, biosecurity practices and challenges of unrecognized backyard poultry flocks maintained in residential areas in Turkey. Despite having been administered exclusively online, the results of the survey showed that there was a significant amount of backyard poultry farming activity in urban and sub-urban areas of Turkey. In general, respondents’ awareness of poultry diseases was low, and the lack of biosecurity measures was notable. Respondents’ lack of knowledge about poultry diseases and biosecurity poses a public health risk, especially in terms of zoonotic diseases, as poultry farming is carried out within residential areas. The findings of this study also highlighted the need for legislation that is specific to backyard poultry owners. Although backyard poultry keeping is a relatively recent phenomenon in Turkey, especially in urban and suburban areas, it is likely to expand over the next decades. Therefore, it is critically necessary to provide comprehensive regulations that refer to considerations such as poultry keeping in residential districts, its biosafety and sanitation, poultry lodging regulations, and poultry welfare.

## Figures and Tables

**Table 1:** The association between respondents’ living environment in terms of backyard poultry practices and challenges.

Question	Response	Respondents’ Environment	Yes (%)	OR *	95% Confidence Interval	*p*-Value
Reasons for keeping poultry	Generating income	Urban	24.4	Reference		
		Sub-urban	13.0	0.78	0.65–0.94	0.012
		Rural	11.1	1.23	1.02–1.49	0.026
Biosecurity awareness	Quarantine of newly arrived chickens	Urban	46.4	Reference		
		Sub-urban	23.1	1.36	1.05–1.77	0.002
		Rural	24.2	0.70	0.52–0.93	0.001
Source of information about poultry healthcare	Poultry veterinarian	Urban	51.4	Reference		
		Sub-urban	23.4	0.76	0.27–2.08	0.000
		Rural	25.2	1.35	0.60–3.01	0.464
	Internet	Urban	39.5	Reference		
		Sub-urban	26.3	4.79	1.72–13.34	0.003
		Rural	34.2	1.77	0.84–3.72	0.132
Use of Veterinary Services	When faced with a disease	Urban	46.6	Reference		
		Sub-urban	29.3	1.57	1.19–2.02	0.001
		Rural	24.1	1.00	0.77–1.30	0.990
Challenges	Lack of regulation	Urban	34.1	Reference		
		Sub-urban	27.5	1.43	1.10–1.87	0.007
		Rural	38.4	2.06	1.60–2.66	0.000
	Animal thief	Urban	30.8	Reference		
		Sub-urban	30.5	1.34	0.97–1.84	0.038
		Rural	38.7	2.19	1.59–3.01	0.000
	Predation	Urban	47.4	Reference		
		Sub-urban	31.8	0.96	0.73–1.25	0.761
		Rural	20.8	1.39	1.28–1.54	0.001

* Odds ratios from multinomial logistic regression.
